# Comparative Study of Larvicidal Activity of Spinel Co_3_O_4_ Nanorods and Entomopathogenic *Metarhizium brunneum* Conidia against *Culex pipiens*

**DOI:** 10.3390/molecules27207035

**Published:** 2022-10-18

**Authors:** Rania A. Mohamed, Wageha A. Mostafa, Lamyaa M. Kassem, Niveen M. Ghazali, Ahmed G. El-Shamy

**Affiliations:** 1 Department of Biology, Deanship of Educational Services, Qassim University, P.O. Box 5888, Unaizah 56219, Saudi Arabia; 2 Entomology Section, Zoology Department, Faculty of Science, Zagazig University, Zagazig 44519, Egypt; 3 Department of Pharmacy Practice, Unaizah College of Pharmacy, Qassim University, P.O. Box 5888, Unaizah 51911, Saudi Arabia; 4 Department of Pharmaceutical Chemistry and Pharmacognozy, Unaizah College of Pharmacy, Qassim University, P.O. Box 5888, Unaizah 51911, Saudi Arabia; 5 Department of Physics, Faculty of Science, Suez Canal University, Ismailia 41522, Egypt

**Keywords:** cobalt oxide (Co_3_O_4_), nanorods, larvicidal, *Culex pipiens*, entomopathogenic fungi, *Metarhizium brunneum*

## Abstract

Herein, we report the synthesis of spinel cobalt oxide nanorods (Co_3_O_4_ NRs) by a modified co-precipitation approach and examine their larvicidal activity against *Culex pipiens.* The structure and morphology of the as-prepared Co_3_O_4_ NRs were emphasized using X-ray diffraction (XRD), Raman spectroscopy, energy dispersive X-ray spectroscopy (EDAX), scanning electron microscopy (SEM), and transmission electron microscopy (TEM). It was found that Co_3_O_4_ nanostructures have a face-centered spinel cubic crystal structure with a mean crystallite size of 38 nm. These nanostructures have a rod like shape with a mean diameter of 30 nm and an average length of 60 nm. The TGA measurements revealed the high stability of the formed spinel cubic structure at 400 °C. The optical behavior indicates the direct transition of electrons through an optical band gap in the range of 2.92–3.08 eV. These unique chemical and physical properties of Co_3_O_4_ NRs enabled them to be employed as a strong agent for killing the *C. pipiens*. A comparison study was employed between the as-prepared Co_3_O_4_ and the entomopathogenic fungus *Metarhizium brunneum* as a control agent of *C. pipiens* larvae. The results revealed that the as-prepared nanorods have higher mortality against *C. pipiens* larvae compared with the well-known *M. brunneum*.

## 1. Introduction

Mosquitoes are responsible for the transmission of many parasites and pathogens that cause severe diseases, including lymphatic filariasis, Rift Valley Fever virus, yellow fever, malaria, and the Zika virus [1]. *C. pipiens* is the main vector of lymphatic filariasis disease (*Wuchereria bancrofti*), causing serious infection to millions of people annually [2]. Moreover, *Culex* spp. is a well-known primary source of annoyance for humans [3]. Recently, the scientific community has worked on using alternative strategies to the harmful chemical insecticide. In this regard, nanotechnology has been introduced as a safe and novel method to control mosquito species. The nanomaterials have gained significant attention from researchers owing to their distinctive physicochemical aspects and remarkable technological applications. In particular, transition metal oxides based nanoparticles of a semiconductor nature such as zinc oxide (ZnO), magnesium oxide (MgO), copper oxide (CuO), titanium dioxide (TiO_2_) and silver oxide (Ag_2_O) have been utilized in agriculture, pharmaceuticals, chemical engineering, and in the biomedical fields. For instance, cobalt oxide nanoparticles (Co_3_O_4_ NPs) of high chemical reactivity, p-type semiconducting, and good magnetic behavior were applied in medicine, supercapacitors, gas sensors, phtotocatalysis, and biological fields as an anticancer, antioxidant, and antimicrobial, with a small amount to overcome the relative toxic side effect [4,5,6]. Tu et al. (2022) have reported the antimicrobial, catalytic degradation activity, and biomedical effect of cobalt oxide nanosheets integrated with silver (Ag/Co_3_O_4_) [7]. Numerous routes have been developed for preparing the nanometal oxides such as sol gel, microwave-assisted reflux, co-precipitation, chemical vapor deposition, chemical spray pyrolysis, and a combustion approach [8,9]. Among the aforementioned techniques, chemical co-precipitation has attracted considerable attention because of its excellent features such as being cost effective, its simplicity in preparation, and its morphological well-defined structure. In the current study, the spinel cobalt oxide nanorods were synthesized by facile chemical approach for larvicidal activity against *C. pipiens*. In general, the nanoparticles can be found in different morphological shapes (i.e., nanosphere, nanoflake, nanocube, etc.) dependence of annealing temperature, electronegativity, and the pH of chemical reaction [10]. In the previous study, cobalt oxide NRs were functionalized as antimicrobials and a control agent for different types of mosquitoes owing to their large surface to volume area and surface plasmons [8,10]. On the other way, the use of entomopathogenic fungi (EPF) including *Metarhizium ansiopliae, M. brunneum*, and *Beauveria bassiana* as biocontrol agents attracted attention from health organizations based on their safety to human health and eco-friendly qualities compared with synthetic insecticides [11,12,13]. Moreover, the insect pathogenic fungus *Metarhizium* infects different developmental stages (eggs, larvae, and adults) of *Aedes, Anopheles*, and *Culex* mosquitoes, causing death [14,15]. EPF have a natural dispersal form known as conidia produced by conidiophores on the surface of the infected hosts. These conidia are resistant to desiccation and remain dormant in the soil for long periods, so they are commonly used for the control of agricultural pests [16]. Here, the strain of *M. brunneum* (petch 3826 CF Andrade) was applied to *C. pipiens* larval control that is characterized by the high yielding of pathogenic conidia [17]. The study revealed that the synthesized Co_3_O_4_ nanorods performed better against *C. pipiens* compared with the utilized biological agent.

## 2. Materials and Methods

### 2.1. Chemicals

Cobalt chloride hexahydrate (CoCl_2_.6H_2_O), glutaraldehyde, cacodylate buffer, osmium tetroxide (OsO_4_), ethanol, acetone, and sodium hydroxide (NaOH) were purchased from Merck and the Alfa-Aesar Company.

### 2.2. Preparation Process

Cobalt oxide (Co_3_O_4_) was prepared by dissolving 3.45 g CoCl_2_.6H_2_O in 60 mL double distilled water under stirring at ambient temperature. Next, 30 mL NaOH solution was carefully added dropwise to the aqueous cobalt chloride with continuous stirring. The resulting precipitate was formed at pH monitored at 9. The powder was subsequently separated using filter paper and then washed many times with distilled water to remove the unreacted salts and residual ions. The product powder was dried in a furnace at 70 °C for 12 h and eventually annealed at 400 °C for 2 h.

### 2.3. Characterization of the Samples

The X-ray diffraction (XRD) (Model; Rigaku Smart Lab.) was carried out at wavelength 1.540 Å to investigate the crystal structure of cobalt oxide through the diffraction angle of 2θ range from 5°–80°. The phase content was recorded by Raman spectroscopy (Horiba Lab RAM HR Evolution). Scanning electron microscopy (SEM; Helios Nanolab. 400) and energy dispersive X-ray analysis (EDX) were examined to describe the surface topology and define the main elemental composition. Transmission electron microscopy (TEM, Hitachi-H-7500) worked at 100 kV was utilized to determine the mean size and particle distribution. Thermogravimetric (TGA, Netzsch Jupiter) was utilized under argon atmosphere to investigate the thermal stability of the nanoparticles. Cobalt oxide thin film was deposited on glass substrates by a spin coater (SpinNXG-P1AC). UV-Vis optical absorbance was measured by a spectrophotometer (Jasco-V-570) for studying the optical properties of the thin film. The infected larvae were prefixed for 2 h in 2.5% glutaraldehyde and then rinsed in a 0.1 M cacodylate buffer (pH 7.2) for 15 min. It was then post-fixed for 1 h in 1% OsO_4_ using the same buffer. The specimens were washed in buffer, dehydrated in an ethanol series, and treated in an acetone solution. After that, the specimens were coated with iridium for the SEM measurements. The mortality data were analyzed with SPSS version 14 using one-way analysis of variance (ANOVA) and Tukey’s HSD tests for pair-wise comparisons. A probit transformation analysis was used to estimate the LC_50_ at a 95% confidence limit of upper and lower values. The rate of mortality was corrected by Abbott’s equation, [18] expressed as:

(1) $$\mathrm{Mortality}\;\left(\%\right)=\frac{T-C}{100-C}\times 100$$

where, T is the dead larvae in treatment, C is the dead larvae in control.

### 2.4. Fungus Culture

The isolate *M. brunneum* petch 3826 CF Andrade (IF#1) was obtained from the Agriculture Research Service (ARSEF) in Ithaca New York (NY), USA. The fungus isolate was cultivated and maintained in a Sabouraud Dextrose Agar yeast (SDAY) medium. The plates were incubated at 28 °C for seven days. Over 2–3 weeks, the fungal culture was utilized to conduct various studies. Conidia were obtained by scraping the culture surface with a sterile loop in 10 mL distilled water. Tween 80 was added at a rate 0.1%. The spore suspension was filtered through a muslin cloth to eliminate mycelia. An upgraded Neubauer haemocytometer was used to calculate the spore count according to the literature [19].

### 2.5. Mosquito Larval Culture

Larvae of *C. pipiens* were brought from Medical Entomology Institution, Cairo, Egypt. They were reared in a chamber insectary at 27 ± 2 °C, 70 ± 5% RH, and 14 L:10 D photoperiod. The adult mosquitoes were kept in cages (30 × 30 × 30 cm) and fed with 10% glucose solution. After a blood meal, the females laid eggs two days later. Egg rafts were gathered and stored in plastic bowls filled with water for hatching. Larvae were fed with commercial fish food (Shulet Carassius) and they were reared in white enamel dishes containing distilled water.

### 2.6. Larvicidal Bioassay

The microbial control agent *M. brunneum* and chemical synthesized Co_3_O_4_ NPs were separately examined against the third instar *C. pipiens* larvae. Firstly, five concentrations (1 × 10^3^, 1 × 10^4^, 1 × 10^5^, 1 × 10^6^ and 1 × 10^7^ spores/mL) of *M. brunneum* were applied. Conidia of each concentration were examined against *C. pipiens* larvae suspended in (0.1%) Tween-80. A group of larvae was treated in distilled water with Tween-80 (0.1%) as a control and by using pure distilled water as the negative control [20]. The NPs were evaluated at varied ratios ranging from 100 ppm to 600 Co_3_O_4_ NPs [21], with minor alterations. For each case, larvae were divided into four replicates. Twenty larvae per replicate were transferred into 200 mL sterilized water in 250 mL plastic cups covered with mosquito netting. All cups were kept at 27 ±2 °C, 70 ± 5% RH with a photoperiod of 14 L: 10 D. Mortality was observed daily for 96 h.

## 3. Results and Discussion

### 3.1. Microstructural Analysis of Co_3_O_4_ Nanorods

**Figure 1:**
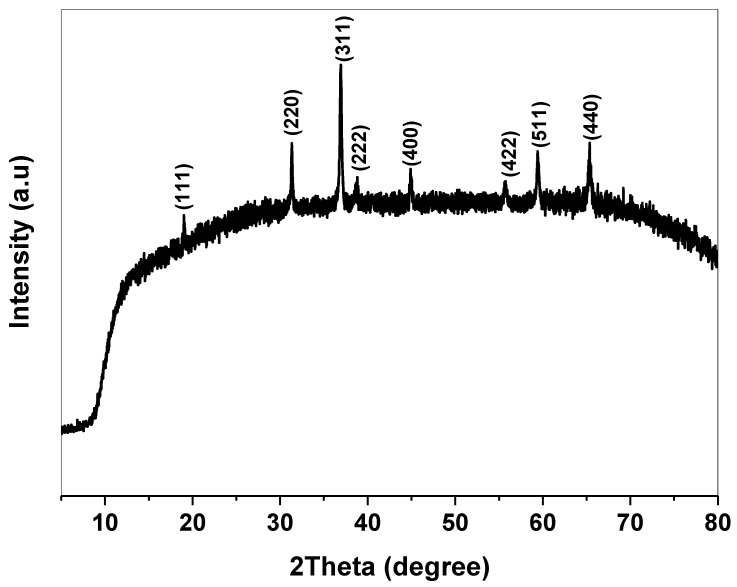
XRD pattern of the spinel Co_3_O_4_ nanoparticles.

Figure 1 shows the XRD pattern of Co_3_O_4_ NRs annealed at 400 C in air for 2 h. The spectrum exhibited a polycrystalline structure of weak crystallinity. The diffraction peaks detected at 2θ 19.07°, 31.40°, 37.02°, 38.80°, 44.95°, 55.61°, 59.46°, 65.32° according to the reflection planes (111), (220), (311), (222), (400), (422), (511), (440) belong to the face-centered cubic phase of spinel Co_3_O_4_ and match with the standard card (JCPDS No. 43-1003, space group Fd3m). No impurities or other phases related to unreacted salts or hydroxyl groups are observed in the pattern, suggesting the high purity of the compound [22]. For more structural analysis, the crystallographic parameters (D,${\;X}_{c},\varepsilon ,\;$𝛿) were calculated from the XRD plot. The average crystallite size (D) was defined using Debye’s Scherrer formula by taking the full width at half maximum (FWHM) of the highest reflected peaks (311) and (440) [23]:

$D=\frac{K\lambda }{\beta \;\cos\theta }\;$; β  is the line broadening at FWHM, k= 0.94 is the shape factor, and λ is the wavelength of incident X-ray radiation = 1.540 Å. The degree of crystallinity ($X_{c})$, lattice strain (𝜀), and dislocation density (𝛿) were also estimated using the equations [24]:

$X_{c}=\frac{0.24}{\beta }\;$,$\;\varepsilon =\frac{\beta }{4\;\tan\theta }\;$, and $\delta =\frac{1}{D^{2}}\;$lines/m^2^, respectively. The summarized results in Table 1 revealed that Co_3_O_4_ NPs of small crystallite size which is attributed to the large surface area. The particle size reduction and the good magnetic characteristics of spinel cobalt oxide allow it to easily penetrate the larval cuticle causing the toxic effect. Furthermore, the high values of the dislocation density (δ) and micro strain (ε) are associated with defects, atom disturbance, and the presence of the vacancies inside the cobalt oxide lattice.

**Figure 2:**
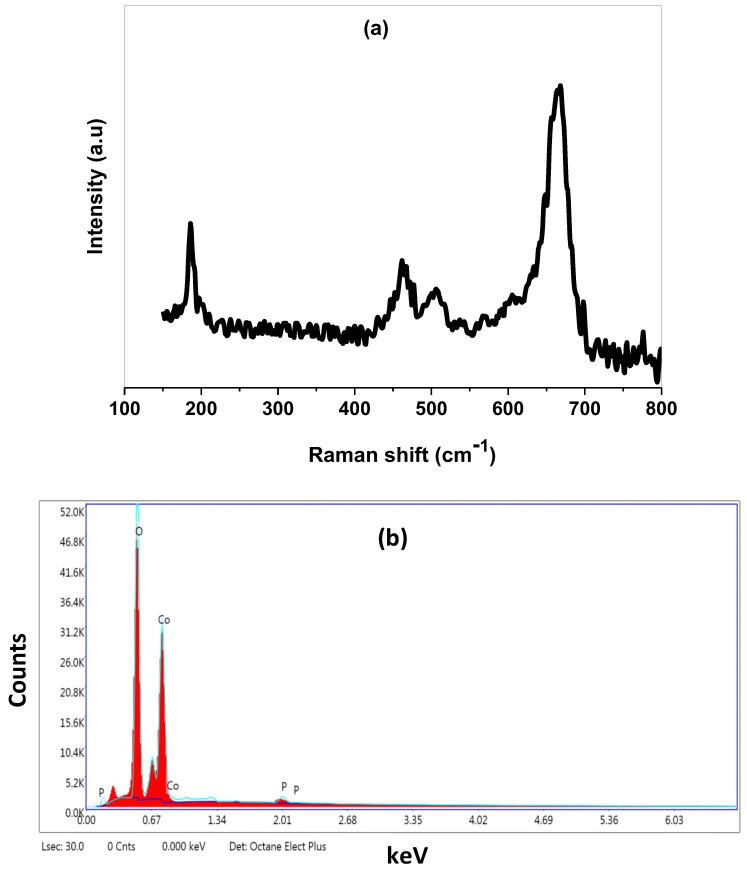
(**a**) Raman spectrum and (**b**) EDX spectrum of Co_3_O_4_ nanoparticles.

**Figure 3:**
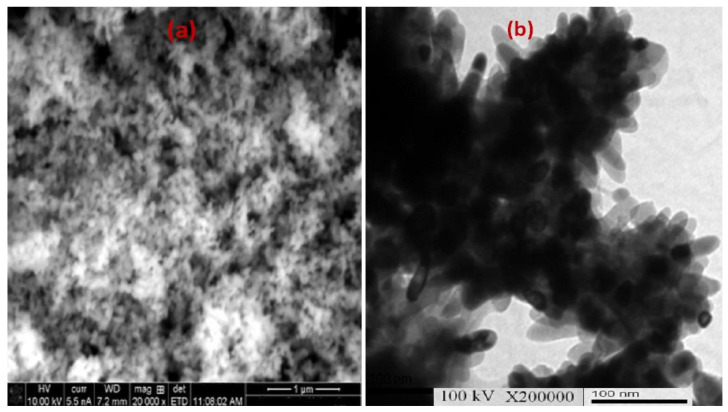
(**a**) SEM and (**b**) TEM micrographs of Co_3_O_4_ nanorods.

To define the molecular structure and phase content, a Raman spectrum was performed at room temperature through the wave number range of 150 to 800 cm^−1^. As illustrated in Figure 2a, there are four active Raman modes located at 667.17 cm^−1^, 506.14 cm^−1^, 462.75 cm^−1^, and 185.05 cm^−1^ which clearly confirm the high quality of the prepared nanomaterial. The strongest peak located at wave number 667.17 cm^−1^ is attributed to the A1g phonon modes of the octahedral (CoO_6_) sites, whereas the peak at 186.976 cm^− 1^ is related to the tetrahedral (CoO_4_) sites [25]. The bands located at 506.14 and 462.75 cm^−1^ are indexed to the second order Raman scattering resulting from phonons. The elemental chemical composition was recorded by EDX spectrum as illustrated in Figure 2b. The EDX spectrum of Co_3_O_4 is_ composed of cobalt (Co) and oxygen (O) elements with a weight percentage of 62.65 and 35.45 wt.%, respectively [10]. The peaks appeared at 0.277 keV and 1.978 keV were assigned to the carbon coated grid and an iridium coated sample was used for increasing its conductivity. No other undesired peaks were detected in the spectrum, which further confirms the high purity of the synthesized spinel compound. The surface morphology, design, and particle distribution were investigated by SEM and TEM spectroscopy. The SEM image (Figure 3a) that describes the surface of Co_3_O_4_ is almost uniformly of sharp tiny rods with high density. Despite the large surface area, little pores were observed which may be related to lattice defects or the presence of vacancies [26,27]. The TEM micrograph described the particles in nanorod shapes of mean diameter ~30 nm and mean length ~60 nm (Figure 3b). Additionally, some of the particles are aggregated together due to the high surface energy and the size confinement effect [28,29].

### 3.2. Thermal and Optical Characteristics

**Figure 4:**
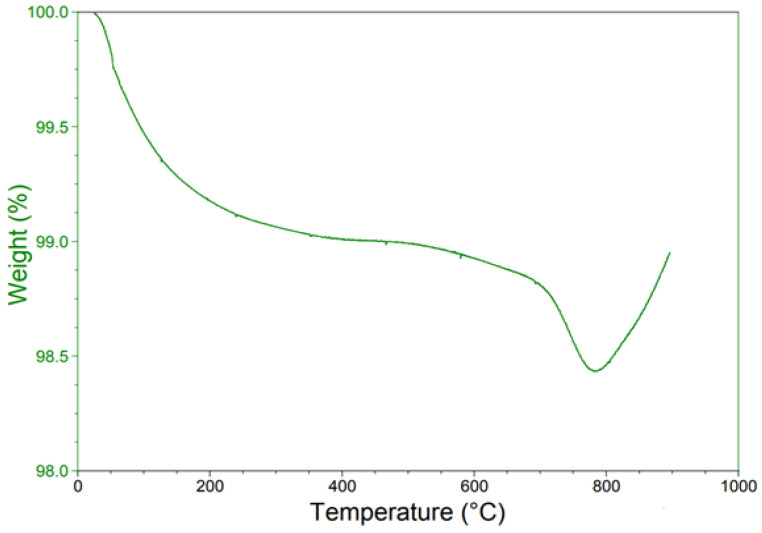
TGA curve of Co_3_O_4_ nanoparticles.

The thermal properties of Co_3_O_4_ NPs were analyzed by thermal gravimetric analysis (TGA) through the temperature range from 50–900 °C, as demonstrated in Figure 4. In the first stage, the weight loss through the temperature range 50–200 °C is the result of the evaporation of water molecules and absorbed moisture. In the second stage (300–400 °C), the TGA curve displayed the weight loss of hydroxyl decomposition and the removal of excess chemical salts. In the temperature range from 450 to 650 °C in the third stage, the spinel compound Co_3_O_4_ showed thermal stability in which there was no loss of weight. In the last stage, up to 700 °C, the weight was suddenly lost, which may be owing to the phase transition [30].

**Figure 5:**
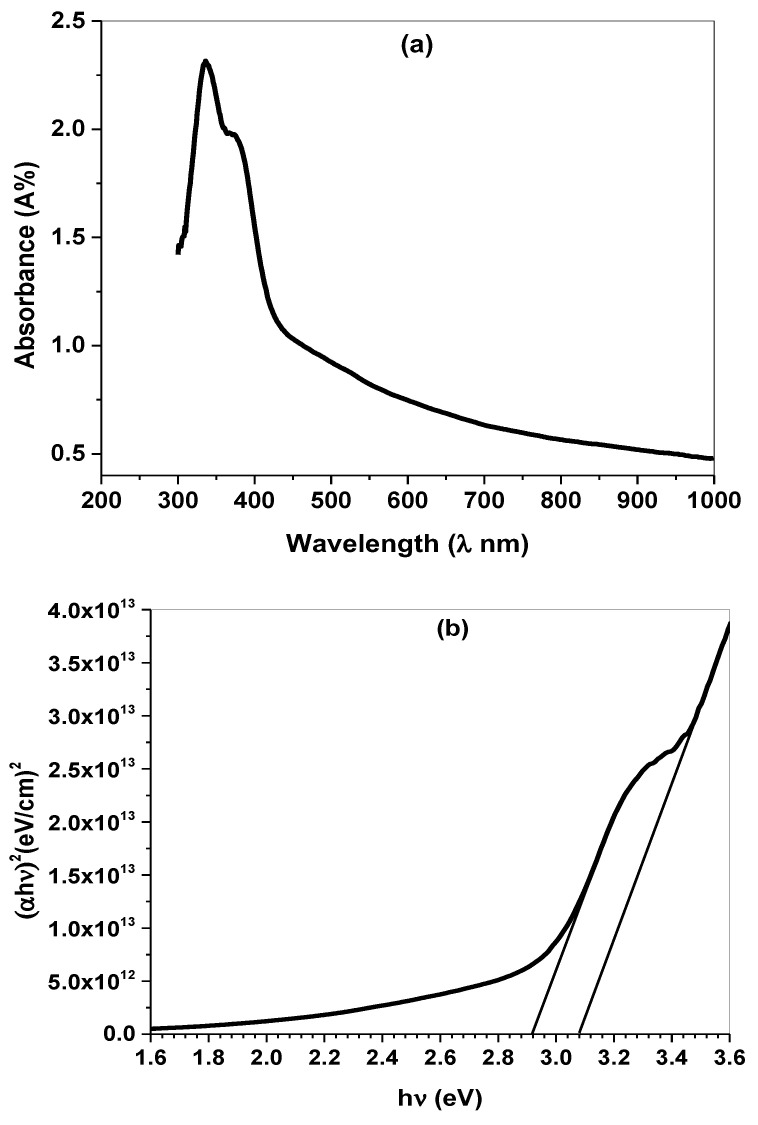
(**a**) Optical absorbance and (**b**) Energy gap of Co_3_O_4_ thin film deposited on glass substrates.

The optical absorption of Co_3_O_4_ thin films was measured within the UV-Vis region, as illustrated in Figure 5a. As can be seen, the spectrum has two absorption bands at 340 and 390 nm, revealing the broad light absorption and the strong interaction between the incident photon energy and the nanoparticles [31]. The presence of two sharp and weak bands is associated with structure disorder as well as generating new energy levels within the band gap. The energy gap $\left(E_{g}\right)$ is an important parameter which identifies the optical characteristics and the type of electron transition. The energy gap of the fabricated thin film was estimated from Tauc’s formula, defined as [30]:

(2) $$\alpha =\frac{A}{h\nu }{\left(h\nu -E_{g}\right)}^{n}$$

where, hν is the incident optical energy, A is the optical absorbance, and α is the optical absorption coefficient expressed as [32,33]:

(3) $$\alpha =\frac{2.303\;A}{t}$$

t is the thickness of the film. The $E_{g}$ of Co_3_O_4_ was evaluated by extending the straight line part of $\;{\left(\alpha h\nu \right)}^{2}$ curve on the Y-axis to the energy on X-axis at $\;{\left(\alpha h\nu \right)}^{2}=0$ (Figure 5b) exhibiting allowed direct transitions at 2.92 and 3.08 eV. The photon energy greater than the band gap means that more electrons transfer from to the conduction band and increase of free charge carriers [32,33].

### 3.3. The Larvicidal Performance of Co_3_O_4_ NPs

**Figure 6:**
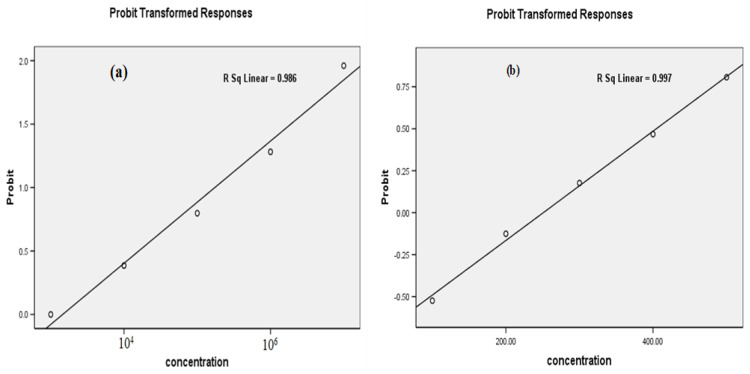
(**a,b**) The probability analysis of mortality of *C. pipiens* mosquito larvae by fungus, *M. brunneum* and Co_3_O_4_ NPs.

The larvicidal performance of Co_3_O_4_ NPs against the third instar larvae of *C. pipiens* was examined. As presented in Table 2, the mortality rate was 100% at the ratio 600 ppm and 30 ± 0.478% at 100 ppm. The difference in mortality at various NPs concentrations was statistically significant (F = 63.393; df = 4; *p* < 0.001) and the LC_50_ value was 250.45 ppm. The probability analysis of *C. pipiens* larval mortality using the nanoparticles was illustrated in Figure 6b [34]. These results are consistent with Kainat et al. (2021) [35], who reported that Co_3_O_4_ nanoparticles have larvicidal potential for *Aedes aegypti* with 67.2 ± 1.7% mortality at 400 ppm compared with MgO NPs of 49.2 ± 8.1% mortality at the same concentration. The impact of cobalt oxide nanoparticles on the treated larvae is a shape-dependent activity. The explanation for this is that when the nanoparticles interact with the body of mosquito larvae, spinel Co_3_O_4_ dissociated into reactive oxygen species and released cobalt ions on the surface of the larvae [36,37]. The reactive oxygen species cause oxidative stress and lipid peroxidation, which inhibits larval growth [38]. Furthermore, nanorods provided an effective direct path for carriers that easily penetrated the mosquito cells leading to larval death [39].

**Figure 7:**
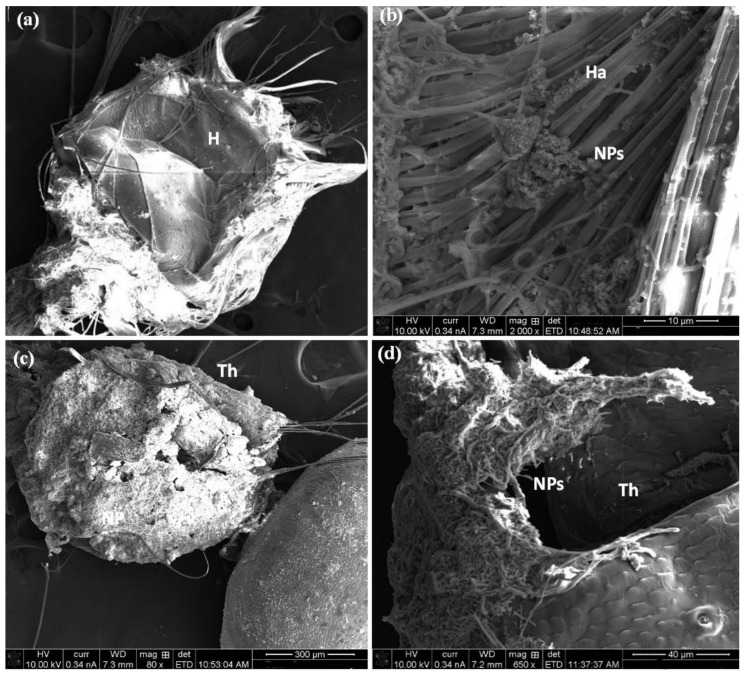
SEM of *C. pipiens* (body parts) exposed to Co_3_O_4_ NPs. (**a**,**b**) show that Co_3_O_4_ adheres to the head region and mouth parts. (**c**,**d**) display the NPs attached to the thoracic cuticle. (Th: thoracic, Cu: cuticle, H: head, Ha: head hair, Mp: mouthparts, NPs: nanoparticles).

**Figure 8:**
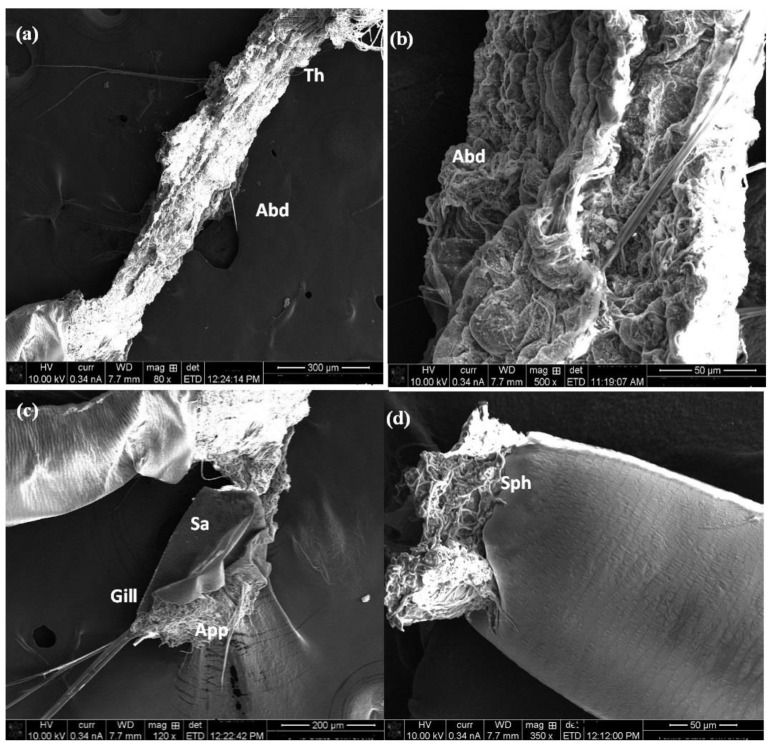
SEM of *C. pipiens* (body parts) exposed to Co_3_O_4_ NPs. (**a**,**b**) show that Co_3_O_4_ adheres to the abdominal cuticle. (**c**,**d**) demonstrate the NPs in the respiratory siphon and gills. (Abd: Abdomen, Cu: cuticle, G: gill, Sa: saddle, Siph; siphon).

A SEM observation showed that Co_3_O_4_ nanorods concentrated on the body surface of the treated larvae (Figure 7). Damage in the head region and accumulation of NPs in the mouth brushes were demonstrated in Figure 7a,b. Also, Figure 7c,d illustrated the high density of Co_3_O_4_ NPs on the thoracic area, especially between the inter-segment, while Figure 8a,b showed deformation of the abdomen cuticle, which is the reason for the rupture of the cuticle wax layer and causes the malfunction of this layer, leading to insect dehydration and finally death. Moreover, the particles were concentrated in the gills and respiratory siphon, causing suffocation, as seen in Figure 8c,d.The toxicity of NPs against mosquito larval instars is related to their ability to penetrate through the exoskeleton, according to earlier studies on mosquito larvae [40,41,42]; when the nanostructure material enters the intracellular space, they bind to sulfur from proteins which leads to the rapid denaturation of organelles. Additionally, the loose membrane permeability and loss of cellular function leads to cell death [43,44].

### 3.4. Entomopathogenic Fungus Infection

**Figure 9:**
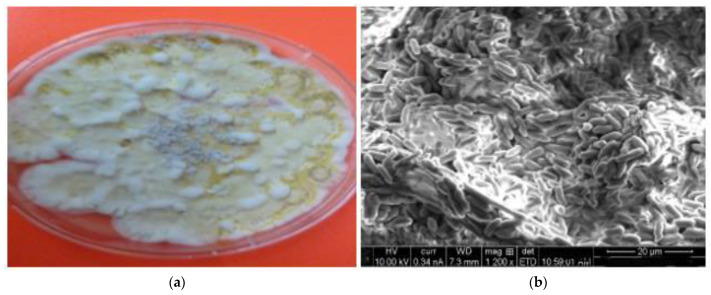
(**a**) Colony growth of *M. brunneum* on SAB media after 14 days of incubation at 27 °C and (**b**) SEM of the fungus culture.

The exposure of the third larval instar of *C. pipiens* to fungus (*M. brunneum*) described in Figure 9a, b revealed larvicidal activity. The treatment of *M. brunneum* demonstrated a significant larval mortality, where, the highest spore concentration (1 × 10^7^) was recorded at larval mortality rates up to 97.5 ± 0.288% at 96 h, while the mortality rate was 50 ± 0.816 at low concentrations. Moreover, the difference in the rates of larval mortality at different concentrations were statistically significant (F = 34.078; df = 4; *p* < 0.000) with LC_50_ value 1.3 × 10^3^ spores/mL (Table 2). The probability analysis of *C. pipiens* larval mortality was illustrated in Figure 6a. The obtained results confirmed that the conidia of the pathogenic terrestrial fungus, *M. brunneum,* has a strong virulence against the larval stage of *C. pipiens*, specifically at the highest concentration. Entomopathogenic fungi have the ability to attack and invade aquatic mosquito stages [45,46]. Other studies show that the conidia of *M. anisopliae* strains are pathogenic against *Aedes, Anopheles*, and *Culex* larvae, and other vectors such as midges and ticks [17,47]. Alkhaibari et al. [48] have reported the positive impact of *M. brunneum* against *C. pipiens* larvae. Additionally, *C. quinquefasciatus* larvae were more susceptible to *M. brunneum* conidia infection than blastosratpore infection due to the larval instars of *C. quinquefasciatus* having a stronger and faster defense reaction to blastospores than conidia [48]. The study showed that the presence of *Culex* larvae close to the water surface expose them to a great amount of hydrophobic conidia [49].

**Figure 10:**
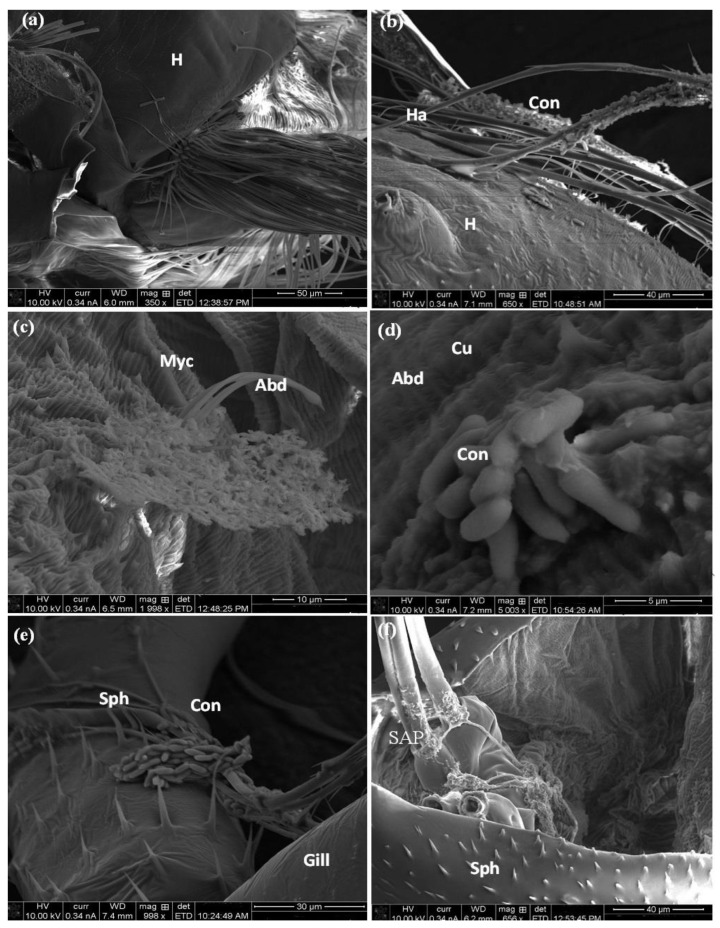
SEM of larval body surface (head, abdomen, and respiratory siphon, gills) of *C. pipiens* treated with fungus, *M. brunneum*. (**a**,**b**) *M. brunneum* adheres to the hair of the head. (**c**,**d**) attachment of *M. brunneum* to the abdominal cuticle and (**e**,**f**) to the respiratory system. (Abd: Abdomen, Co: conidia, Cu: cuticle, Gill: gills, H: head, Ha: head hair, Siph: siphon, SAP: spiracular apparatus).

In the SEM micrograph observation, infected larvae showed that the fungal spores of *M. brunneum* are attached to the surface of the larval cuticle of *C. pipiens* at various body parts (Figure 10) at 48 h post-treatment. These conidia stuck to the head hair (Figure 10a,b). In addition, the fungus’ mycelium penetrated the abdomen cuticle (Figure 10c,d.) Moreover, conidia penetrated and germinated in the siphon and gills leading to blocking the respiratory opening and larval suffocation (Figure 10e,f). These observations were coincident with Alkhaibari et al. [45], who revealed that *M. brunneum* blastospores adhered to the *Aedes aegypti* cuticle surface 24 h after treatment. Furthermore, conidia adhesion to *C. quinquefasciatus* larvae was shown to be higher on the siphon and head areas, particularly around the mouthparts [15]. Furthermore, hydrophobic lipids in the external epicuticular wax layer can play a vital role in the attachment and germination of fungal propagules [50]. Fungi with hydrophobic conidia allow hyphal growth into the tracheal system causing suffocation to the host and leading to its death [51]. Previous studies investigate the histopathological influence of entomopathogenic fungi on different insect structures, which indicated that the most relevant site of fungal attachment is the insect cuticle [44]. EPF exhibit their effect on the insect cuticle both mechanically, by penetration, as well as through chemical lysis of the cuticle and the whole body tissues by the action of chitinase, protease, and lipase enzymes [52].

To elucidate whether the effect of Co_3_O_4_ nanorods is beneficial as an effective larvicidal nanomaterial, we compared the synthesized nanorods results with the fungus. The mortality rates were estimated to be 100% for NRs and 97.5% for the fungus. Therefore, the Co_3_O_4_ NRs were more effective in the control of mosquito larvae.

## 4. Conclusions

Spinel cobalt oxide nanorods with aspect ratio (length/diameter = 2) were successfully prepared by a simple chemical approach. These nanorods showed high thermal stability and optical band gap within the visible region (2.98–3.08 eV). These unique properties enabled the as-prepared cobalt oxide to be used as an efficient agent against *C. pipiens.* The results confirmed that the nanorods have the ability to get rid of all of the larvae with high efficiency compared to the fungus. Therefore, one may conclude that a new strong agent based on Co_3_O_4_ NRs was developed to suppress mosquito larvae.

## Figures and Tables

**Table 1:** The crystallographic parameters of Co_3_O_4_ NRs.

The Nanoparticles	Crystallographic Parameters				
**Co_3_O_4_**	**FWHM**	**D (nm)**	δ $\times 10^{-4}\;{\left(\mathrm{nm}\right)}^{-2}$	$X_{c}$	$\varepsilon \times 10^{-3}$
	0.23075	38.00	7.00	59.55	12.00

**Table 2:** Toxic effect of fungus, *M. brunneum*, and Co_3_O_4_ NPs against 3rd larval instar of *C. pipiens*.

Test Samples	Conc.	% Mortality Percentage, Mean ± SE	LC_50_ (LCL–UCL)	Regression Equation	X^2^
Co_3_O_4_	**ppm**		250.45 (209.20:285.72 ppm)	Y = −0.97 + (X × 0.007)	0.201
	100	30 ± 0.478			
	200	45 ± 0.250			
	300	57 ± 0.478			
	400	68 ± 1.080			
	500	79 ± 0.250			
	600	100 ± 0.00			
*Metarhizium brunneum*	**spores/mL**		1.3 × 10^3^ (5.86 × 10^2^:2.31 × 10^3^ spores/mL)	Y = −51.5 + (X × 0.5)	0.906
	1 × 10^3^	50 ± 0.816			
	1 × 10^4^	65 ± 0.707			
	1 × 10^5^	78.75 ± 0.629			
	1 × 10^6^	90 ± 0.707			
	1 × 10^7^	97.5 ± 0.288			

LC_50_: lethal concentration that kills 50% of the exposed *C*. *pipiens* larvae; UCL: upper confidence limit; LCL: lower confidence limit; *X*^2^ = Chi-square, *p* < 0.05, significance level, Mean value of five replicates, (SE) standard error. Control (distilled water), nil mortality.

## Data Availability

Not applicable.

## Sample Availability

Samples of the compounds are available from the authors.
